# Antioxidants and α-glucosidase inhibitors from *Neptunia oleracea* fractions using ^1^H NMR-based metabolomics approach and UHPLC-MS/MS analysis

**DOI:** 10.1186/s12906-018-2413-4

**Published:** 2019-01-07

**Authors:** Soo Yee Lee, Ahmed Mediani, Intan Safinar Ismail, Maulidiani, Faridah Abas

**Affiliations:** 1 Laboratory of Natural Products, Institute of Bioscience, Universiti Putra Malaysia, 43400 Serdang, Selangor Malaysia; 2 Atta-ur-Rahman Institute for Natural Product Discovery, Universiti Teknologi MARA, Puncak Alam Campus, 42300 Bandar Puncak Alam, Selangor Malaysia; 3 Department of Chemistry, Faculty of Science, Universiti Putra Malaysia, 43400 Serdang, Selangor Malaysia; 4 Department of Food Science, Faculty of Food Science and Technology, Universiti Putra Malaysia, 43400 Serdang, Selangor Malaysia

**Keywords:** *Neptunia oleracea* fractions, ^1^H NMR-based metabolomics, UHPLC-MS/MS, Phenolics, Diabetes

## Abstract

**Background:**

*Neptunia oleracea* is a plant cultivated as vegetable in Southeast Asia. Previous works have revealed the potential of this plant as a source of natural antioxidants and α-glucosidase inhibitors. Continuing our interest on this plant, the present work is focused in identification of the bioactive compounds from different polarity fractions of *N. oleracea*, namely hexane (HF), chloroform (CF), ethyl acetate (EF) and methanol (MF).

**Methods:**

The *N. oleracea* fractions were obtained using solid phase extraction (SPE). A metabolomics approach that coupled the use of proton nuclear magnetic resonance (^1^H NMR) with multivariate data analysis (MVDA) was applied to distinguish the metabolite variations among the *N. oleracea* fractions, as well as to assess the correlation between metabolite variation and the studied bioactivities (DPPH free radical scavenging and α-glucosidase inhibitory activities). The bioactive fractions were then subjected to ultra-high performance liquid chromatography tandem mass spectrometry (UHPLC–MS/MS) analysis to profile and identify the potential bioactive constituents.

**Results:**

The principal component analysis (PCA) discriminated EF and MF from the other fractions with the higher distributions of phenolics. Partial least squares (PLS) analysis revealed a strong correlation between the phenolics and the studied bioactivities in the EF and the MF. The UHPLC-MS/MS profiling of EF and MF had tentatively identified the phenolics present. Together with some non-phenolic metabolites, a total of 37 metabolites were tentatively assigned.

**Conclusions:**

The findings of this work supported that *N. oleracea* is a rich source of phenolics that can be potential antioxidants and α-glucosidase inhibitors for the management of diabetes. To our knowledge, this study is the first report on the metabolite-bioactivity correlation and UHPLC–MS/MS analysis of *N. oleracea* fractions.

## Background

Diabetes mellitus is a metabolically complicated disease in which the patient experiences a high blood glucose level. Over the past few decades, the occurrence of diabetes mellitus has increased drastically. Globally, the number of diabetic cases in adult population has increased from 108 million in 1980 to 422 million in 2014 [1]. Among diabetic patients, the majority suffer from type 2 diabetes, which is associated with insulin insensitivity in the body tissue. The impaired glucose metabolism leads to excessive production of reactive oxygen species (ROS), enhances oxidative stress in the body and ultimately leads to complications such as cardiovascular disease, retinopathy, nephropathy and impaired wound healing. One of the therapeutic approaches for diabetes management is to slow down the absorption of postprandial glucose by inhibiting carbohydrate hydrolyzing enzymes, such as α-glucosidase [2]. However, the currently available α-glucosidase inhibitors including acarbose, voglibose and miglitol produce gastrointestinal side effects [3]. Hence, efforts are being made to search for antioxidants and α-glucosidase inhibitors from natural resources for the management of diabetes and its complications.

*Neptunia oleracea* is a plant cultivated as vegetable in Southeast Asia. It has a pleasant flavor and can be eaten raw or cooked. Proximate analysis of *N. oleracea* has shown that this plant is a good source of crude protein, crude fiber and important minerals, such as potassium and calcium [4]. Furthermore, this plant has been reported to contain a high level of pro-vitamin A carotenoids, which are important in preventing vitamin A deficiency [5]. Studies have revealed that this plant exhibited several biological activities, including antiulcer [6], hepatoprotective [7], antiviral [8], 5α-reductase inhibitory [9], analgesic and antiinflammatory [10] activities. Besides, to identify antioxidants and α-glucosidase inhibitors from natural resources, *N. oleracea* has been studied by our research group for its free radical scavenging and α-glucosidase inhibitory properties. The results have shown that this plant is a prominent source of free radical scavengers and α-glucosidase inhibitors; with phenolics as the potential candidates for these active compounds [11, 12].

Most of the previous studies of *N. oleracea* focused on the crude extracts and the metabolites contributing to these pharmacological properties were remained unassigned [6–9]. Often, the metabolite profile of crude extracts is complicated due to the large amount of metabolites that could be present, making the identification process challenging. One of the approach to facilitate the analysis and make the identification of the important metabolites more effective is to fractionate the crude extract by different polarity. Hence, in this study, the crude extract of *N. oleracea* was fractionated according to polarity using solid phase extraction (SPE). The metabolite variation among these fractions as well as the correlation between the metabolites and the studied bioactivities (DPPH free radical scavenging and α-glucosidase inhibitory activities) were assessed using a ^1^H NMR-based metabolomics approach. The potential bioactive constituents present in the bioactive fractions were profiled and identified using ultra-high performance liquid chromatography tandem mass spectrometry (UHPLC–MS/MS). These procedures help to highlight the bioactive fractions of *N. oleracea* for the DPPH free radical scavenging and α-glucosidase inhibitory activities, and to reveal the bioactive compounds, as continuation of our previous efforts on the investigation of *N. oleracea* as a source of antioxidant and α-glucosidase inhibitors.

## Methods

### Chemicals and reagents

Phosphate buffer, glycine, α-glucosidase enzyme, *p*-nitrophenyl-α-D-glucopyranose (PNPG) and 2,2-diphenyl-1-picrylhydrazyl (DPPH) were purchased from Sigma-Aldrich (Hamburg, Germany). Analytical grade solvents including absolute ethanol, hexane, chloroform, ethyl acetate and methanol were supplied by Merck (Darmstadt, Germany). The NMR chemicals including deuterated dimethyl sulfoxide-*d*_*6*_ (DMSO-*d*_*6*_) and trimethylsilyl propionic acid-*d4* sodium salt (TSP) were also supplied by Merck (Darmstadt, Germany). The LC/MS grade acetonitrile and formic acid were purchased from Merck (Darmstadt, Germany). Water was prepared using a Milli-Q purification system.

### Plant materials

*Neptunia oleracea* was planted in Universiti Putra Malaysia Agricultural Park by spreading the stems onto a pond. The plant was authenticated by an in-house Botanist (Dr. Shamsul Khamis) of the Institute of Bioscience, Universiti Putra Malaysia and the voucher specimen (SK2516/14) was deposited in the Herbarium Biodiversity Unit of the institute.

### Sample drying and extraction

The optimized conditions of drying and extraction (freeze drying and sonication with absolute ethanol) were selected based on previous works on *N. oleracea* [11, 12]. The three-months-old plants were harvested. Immediately after the harvest, the leaves were separated from the stems of the plants. The leaves were cleaned with water and then kept in freezer at − 80 °C. The frozen leaves were then lyophilized until constant weight. The dried leaves were then powdered and used for extraction. To produce the crude extract, 4 g of powdered samples were soaked in 100 mL of absolute ethanol and subjected to sonication for 1 h in an ultrasonic bath sonicator (Branson, 1418510E-MTH model, Danbury, USA). The mixtures were then centrifuged at 13, 000 rpm for 30 min to separate the supernatant and the precipitates. The supernatant was collected and concentrated. Six individual extractions were performed to obtain 6 replications of samples.

### Fractionation of crude extract

The crude extracts were subjected to solid phase extraction (SPE) in order to obtain fractions with different polarities. A weight of 1 g crude extract was premixed with 2 g of silica powder and loaded onto the SPE column cartridge (Isolute®, Mid Glamorgan, UK). The crude extract was then eluted consecutively with hexane, chloroform, ethyl acetate and methanol to yield hexane (HF), chloroform (CF), ethyl acetate (EF) and methanol (MF) fractions. Each solvent was allowed to pass through the cartridge until the eluent obtained was colorless. Each fraction collected was concentrated using rotary evaporator and finally lyophilized and kept in chiller at 4 °C for further analysis. Six individual SPE were performed in order to obtain the 6 replicates.

### Total phenolic content (TPC)

The TPC was determined using a previously reported procedure [13]. Samples were prepared by dissolving the fractions in DMSO. A total of 20 μL of samples and 100 μL of Folin-Ciocalteu reagent were mixed in the 96-well plates and incubated for 5 min. Later, 80 μL of 7.5% sodium carbonate solution was added. The plate was then incubated in the dark for 30 min and checked for the absorbance at 765 nm using a microplate reader (SPECTRAmax PLUS, Sunnyvale, CA, USA). The analysis was performed in 3 determinations for every sample. A standard curve of gallic acid was obtained to calculate the TPC and the results were expressed in μg GAE/mg extract.

### DPPH free radical scavenging assay

The DPPH free radical scavenging activity of the fractions was evaluated according to the method previously described [14], with some modifications. The samples were prepared in various concentrations by serial dilution of stock solutions (1.0 mg/mL) prepared in DMSO. The test samples (50 μL) was mixed with DPPH (100 μL) in the wells. A set of wells was designated as reagent blank where the test sample was replaced by equal volume of sample solvent. The absorbance was then checked at 515 nm using microplate reader after incubation of the plate in dark for 30 min. The percentage of inhibition was calculated as % inhibition = [(A_B_-A_S_)/A_B_] × 100, where A_B_ and As are the absorbance of reagent blank and tested samples, respectively. The analysis was performed in 3 determinations for every sample. The results are expressed as IC_50_ value in μg/mL. Quercetin was used as positive control in this assay.

### α-Glucosidase inhibition assay

The inhibitory activity on α-glucosidase was assayed according to the previously described procedure [15], with some modifications. Samples from each fraction were prepared using DMSO at 1 mg/mL concentration as stock and further diluted to 8 serial dilutions using DMSO containing buffer. A total of 10 μL test sample, 130 μL of 30 mM phosphate buffer and 10 μL of α-glucosidase enzyme (prepared in 50 mM phosphate) were pre-incubated in the 96-well plate for 5 min at room temperature. A set of wells was designated as negative control where the test sample was replaced by equal volume of sample solvent. Next, 50 μL of PNPG substrate were added and the plate was further incubated for 15 min. The reaction was discontinued by addition of 50 μL of 2 M glycine (pH 10) and the absorbance was then read at 405 nm using a microplate reader. The percentage of inhibition was calculated as % inhibition = [(A_n_-A_s_)/A_n_] × 100%, where A_n_ and A_s_ are the absorbance values of the negative control and test samples, respectively. The analysis was performed in 3 determinations for every sample. Results were expressed as IC_50_ values in μg/mL. Quercetin was used as positive control in this assay.

### NMR measurement

The NMR measurements were carried out based the method reported by Mediani et al. [16], with some modifications. Five milligrams of samples were mixed with 500 μL DMSO-*d*_*6*_ (containing 0.1% TSP). The mixture was then sonicated for 15 min and centrifuged at 13,000 rpm for 10 min. The supernatant was transferred to NMR tubes. The ^1^H NMR spectra were acquired using a 500 MHz Varian INOVA NMR spectrometer running at a frequency of 499.887 MHz and a temperature of 26 °C. The time to acquire each spectrum with presat setting was 3.54 min, comprising of 64 scans with a width of 20 ppm. Phase and baseline of all the ^1^H-NMR spectra were corrected using Chenomx software v. 8.1 (Alberta, Canada).

### Ultra-high performance liquid chromatography tandem mass spectrometry analysis (UHPLC-MS/MS) of bioactive fractions

The UHPLC-MS/MS analysis of the bioactive fractions was carried out according to Zilani et al. [17], with modifications. The UHPLC was performed using a reversed-phase UHPLC Thermo system fitted with a Hypersil Gold, Thermo C_18_ column (2.1 mm × 100 mm, 1.9 μm). The mobile phase was 0.1% formic acid in water (solvent A) and 0.1% formic acid in LC–MS grade acetonitrile (solvent B). The analysis time was 33 min, and the flow rate was 250 μL/min. The injection volume was set to 5 μL. The programmed gradient proceeded using the following sequence for solvent B: 5% at 0 min, 15% at 6 min, 20% at 15 min, 20% at 18 min, 25% at 28 min, 100% at 30 min, 100% at 33 min. The samples were prepared by dissolving 4 mg of fraction in 2 mL of HPLC grade methanol, which was then centrifuged and filtered through a 0.22 µm nylon membrane into a 2-mL screw-capped sample vial. Mass spectra identification was attained using the Thermo Finnigan model (San Jose, CA, USA) Thermo Scientific™ Q Exactive™ Hybrid Quadrupole-Orbitrap mass spectrometer with an ESI source coupled to a Surveyor UHPLC binary pump and auto-sampler. This LC–MS/MS system combines quadrupole precursor ion selection with high resolution accurate mass (HRAM) Orbitrap detection and it is useful for targeted and untargeted screening and the identification of unknown compounds. The collision-induced dissociation (CID) energy was at 35%. This system was monitored by Xcalibur 2.2 and Mass Frontier 7.0 software. The negative and positive ion mass spectra were obtained in full ion scan mode (200–1700 amu) at a scan rate of 0.5 Hz. The most abundant ions in each scan were selected and subjected to MS/MS analysis.

### Data analysis

Minitab software version 16 (Minitab Inc., State College, PA, USA) and GraphPad InStat version 2.02 (San Diego, USA) were used for the analysis of TPC, DPPH and α-glucosidase inhibition assay result data. Results were expressed as mean ± SD of 6 replicates. For multivariate data analysis (MVDA), the ^1^H-NMR spectra were bucketed and converted to ASCII files using Chenomx software. A total of 246 integrated regions were obtained after binning the region δ 0.5–10.0 with a width of δ 0.04. The residual signal of DMSO was excluded at δ *2*.42–2.62. The data file was then imported into SIMCA-P software version 13.0 (Umeå, Sweden) for MVDA. Principal component analysis (PCA) and partial least-square analysis (PLS) were performed with Pareto scaling method. Pearson’s correlation was performed using MetaboAnalyst 2.5, a free metabolomics data analytical tool available online (http://www.metaboanalyst.ca).

## Results

### Visual inspection of ^1^H NMR spectra of *Neptunia oleracea* fractions

**Fig. 1:**
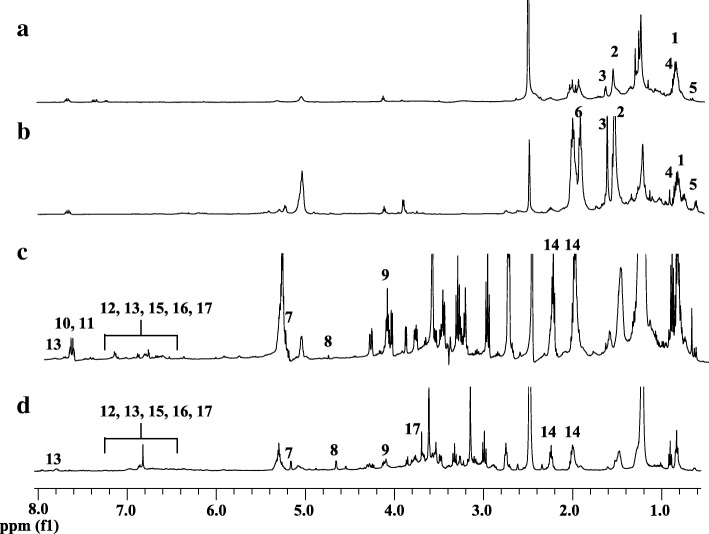
The representative ^1^H NMR spectra of HF (**a**), CF (**b**), EF (**c**) and MF (**d**) of *N. oleracea*. 1, fatty acids; 2, alanine; 3, leucine; 4, valine; 5, oleanolic acid; 6, phytosterol; 7, α-glucose; 8, β-glucose; 9, fructose; 10, quercetin derivatives; 11, kaempferol derivatives; 12, myricetin derivatives; 13, apigenin derivatives; 14, catechin; 15, gallic acid; 16, 3,4-*O*-dimethylgallic acid; 17, caffeic acid

The *N. oleracea* fractions (HF, CF, EF and MF) were subjected to ^1^H NMR analysis and the ^1^H NMR spectra were examined to identify the metabolites present in each fraction. The representative ^1^H NMR spectra of the fractions are shown in Fig. 1. Several classes of metabolites were identified from the fractions, including amino acids, fatty acids, phytosterols, sugars, flavonoids, triterpenes and phenolic acids. The chemical shifts of the identified metabolites are presented in Table 1. Primary metabolites were identified using the Chenomx database, whereas secondary metabolites were identified by comparison with reported data [18–24].

**Table 1:** ^1^H NMR signals of identified metabolites in *N. oleracea* fractions

Metabolites	^1^H NMR signals	Fractions
Phytosterol	0.68 (s), 2.02 (m), 5.3 (m)	CF
Fatty acids	0.88 (m), 1.24–1.35 (m), 1.66 (m), 2.34 (t, *J* = 7.5 Hz)	HF, CF
Alanine	1.48 (d, *J* = 6.5 Hz)	HF, CF
Leucine	0.95 (t, *J* = 6.0 Hz), 1.7 (m)	HF, CF
Valine	0.98 (d, *J* = 6.5 Hz), 2.24 (m)	HF, CF
α-Glucose	5.16 (d, *J* = 3.5 Hz)	EF, MF
β-Glucose	4.62 (d, *J* = 9.5 Hz)	EF, MF
Fructose	4.16 (d, *J* = 6.5 Hz)	EF, MF
Oleanolic acid	0.70 (s), 0.74 (s), 0.81 (s), 0.87 (s), 0.95 (s), 1.10 (s)	HF, CF
Quercetin derivatives	6.36 (s), 6.93 (d, *J* = 9.5 Hz), 7.66 (dd, *J* = 2.3, 9.5 Hz), 7.71 (d, *J* = 2.0 Hz)	EF, MF
Kaempferol derivatives	6.05 (d, *J* = 1.5 Hz), 6.41 (d, *J* = 2.0 Hz), 6.85 (d, *J* = 8.0 Hz), 7.71 (d, *J* = 8.6 Hz)	EF, MF
Myricetin derivatives	6.26 (d, *J* = 2.3 Hz), 6.38 (s), 6.90 (d, *J* = 2.0 Hz)	EF, MF
Apigenin derivatives	6.55 (s), 6.93 (d, *J* = 8.0 Hz), 7.92 (d, *J* = 7.5 Hz)	EF, MF
Catechin	2.03 (dd, *J* = 16.6 Hz), 2.25 (dd, *J* = 15, 3.5 Hz), 3.79 (m), 5.88 (d, *J* = 2 Hz), 6.58 (d, *J* = 2 Hz), 6.69 (d, *J* = 8.9 Hz)	EF, MF
Gallic acid	6.84 (s)	EF, MF
3,4-*O*-dimetgylgallic acid	3.78 (s), 6.84 (s)	EF, MF
Caffeic acid	6.86 (s)	EF, MF

*HF* hexane fraction; *CF* chloroform fraction; *EF* ethyl acetate fraction; *MF* methanol fraction

In the aliphatic region (δ 0.5–3.0) of the HF and the CF, signals of fatty acids and some amino acids, including leucine, alanine and valine were detected. The methyl signals (δ 0.8–0.9) assigned to a triterpene, namely oleanolic acid was also found in these fractions. Besides, signals of phytosterol were also detected in the CF. In contrast, the sugar signals (δ 3.0–5.0) were more prominent in the EF and the MF. Sugars such as α-glucose, β-glucose, and fructose were detected in both fractions. In the aromatic region (δ 5.5–8.5), signals belonging to flavonoids were also detected in these polar fractions. These signals were assigned for catechin and derivatives of quercetin, kaempferol, myricetin, and apigenin. Furthermore, the signals of three phenolic acids (caffeic, gallic and 3,4-*O*-dimethylgallic acids) were also observed in these fractions. These flavonoids and phenolic acids are the metabolites that were identified in previous works to be potential and important antioxidants and α-glucosidase inhibitors in *N. oleracea* [11, 12]. In addition, the signals for the anomeric protons of the arabinosyl, rhamnosyl and glucosyl moieties of the flavonoid derivatives were more obvious in the EF and MF as compared to the crude extracts and were found at δ 5.27 (d, *J* = 6.8 Hz), δ 5.15 (d, *J* = 1.5 Hz) and δ 5.33 (d, *J* = 10.2 Hz), respectively.

Visual inspection of the ^1^H NMR spectra revealed both qualitative and quantitative variations among the different fractions. The metabolites, including phytosterol, triterpene, fatty acids and amino acids resided mainly in the HF and CF, while the sugars and phenolics were more likely to reside in the EF and MF. However, for unbiased interpretation of the results, the ^1^H NMR spectral data of these fractions were further subjected to MVDA. The unsupervised MVDA, PCA, was used to differentiate these fractions based on their metabolite contents and to highlight the metabolites that contributed to the differentiation.

### Metabolic classification of *Neptunia oleracea* fractions

**Fig. 2:**
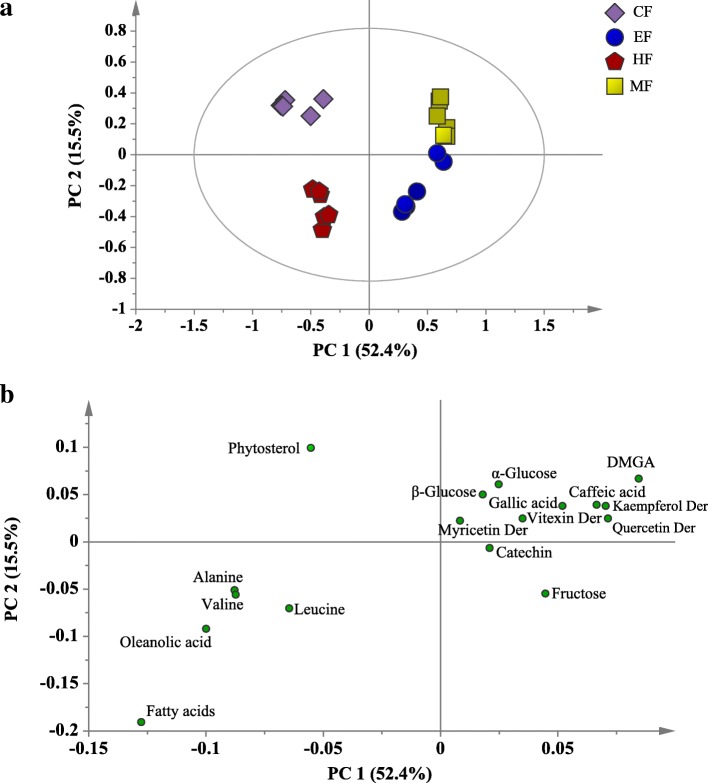
The PCA score (**a**) and loading (**b**) plots of different fractions of *N. oleracea*. HF, hexane fraction; CF, chloroform fraction; EF, ethyl acetate fraction; MF, methanol fraction; Der, derivatives; DMGA, 3,4-*O*-dimethylgallic acid

**Fig. 3:**
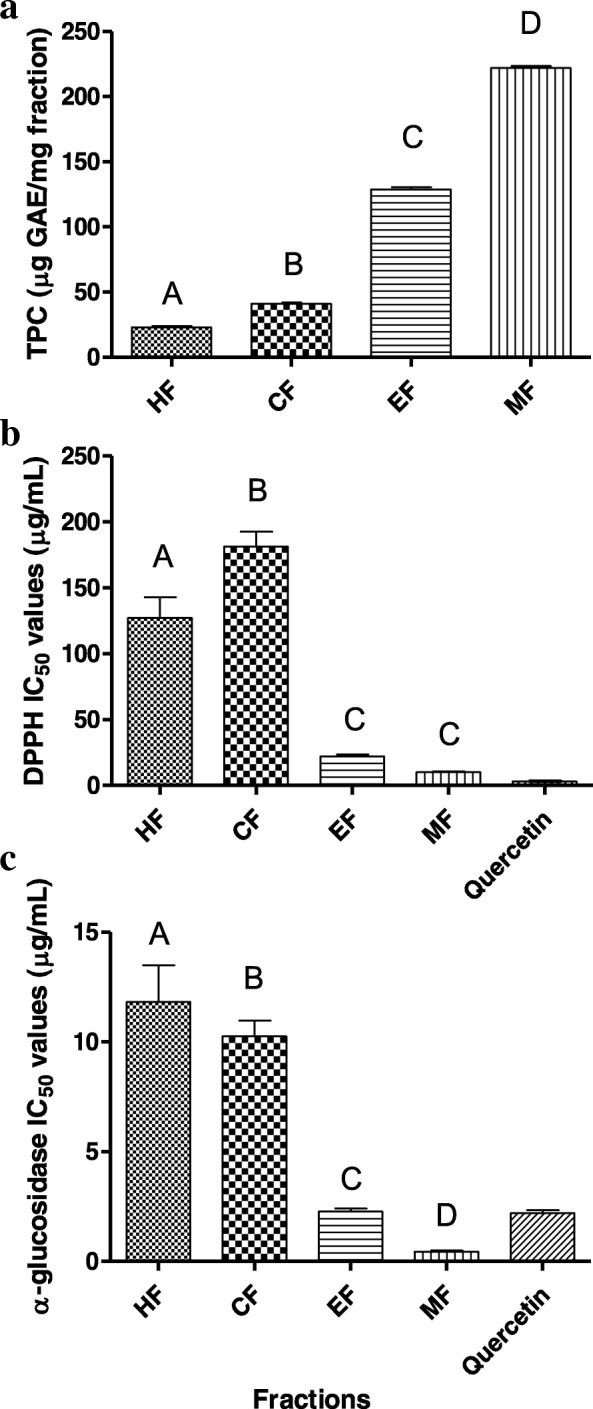
Total phenolic content (**a**), DPPH free radical scavenging (**b**) and α-glucosidase inhibition (**c**) of different fractions of *N. oleracea*. The values are the means ± standard deviations. Means with different letters are significantly different (*P* < 0.05). HF, hexane fraction; CF, chloroform fraction; EF, ethyl acetate fraction; MF, methanol fraction

The PCA score and loading plots of the ^1^H NMR data from the *N. oleracea* fractions are shown in Fig. 2. The score plot shows a clear discrimination of the more polar fractions (MF and EF) from the less polar fractions (CF and HF) by PC1, which contributed to most of the variance (52.4%). The more polar fractions are clustered on the positive side of PC1, while the less polar fractions are located on the other side. In addition, the MF and EF samples are projected close to one another in the score plot. This finding indicates that the EF and MF are closely related in their phytochemical contents and might contain the same metabolites in different intensities. From the loading plot, it is obvious that the less polar fractions (HF and CF) were separated from the other fractions by their higher contents of phytosterol, oleanolic acid, amino acids and fatty acids. Meanwhile, the EF and MF were discriminated from the HF and CF by their higher content of sugars and identified phenolics. These findings support the observation obtained via the visual inspection of the ^1^H NMR spectra. The presence of the identified phenolics in the EF and MF might contribute to the potent DPPH free radical scavenging and α-glucosidase inhibitory activities of the EF and MF. This can be observed in the results of the TPC, DPPH free radical scavenging and α-glucosidase inhibitory activities of the fractions, which showed significant TPC and inhibitions of the DPPH free radical and the α-glucosidase enzyme by the EF and MF (Fig. 3). In order to confirm the contribution of the phenolics to the bioactivities of EF and MF, the correlation was assessed between the identified phenolics and the studied bioactivities.

### Correlation between phenolics and bioactivities of *Neptunia oleracea* fractions

**Fig. 4:**
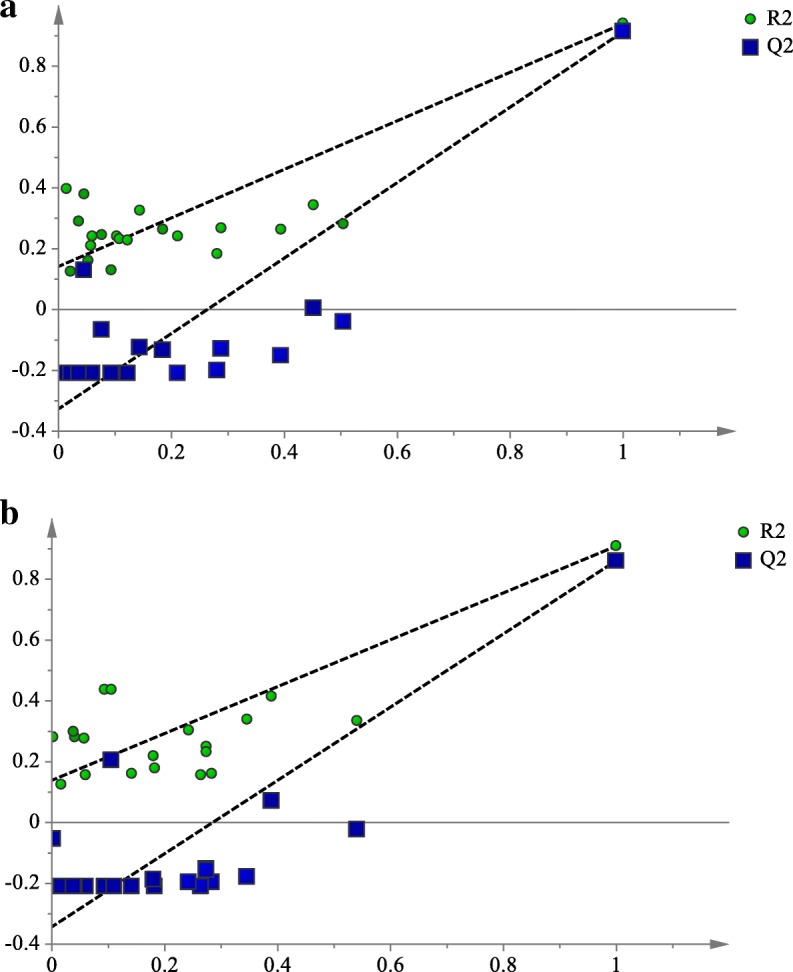
Permutation plots of PLS model describing the R^2^ and Q^2^ Y-intercepts for DPPH free radical scavenging (**a**) and α-glucosidase inhibitory (**b**) activities of *N. oleracea* fractions

The PLS model was constructed for the correlation evaluation of phenolics with the bioactivities of EF and MF, where the NMR chemical signals were input as X variables and the 1/IC_50_ values of the DPPH radical scavenging and α-glucosidase inhibitory activities were the Y variables. The resulted PLS model was validated by goodness of fit, prediction of Y and a permutation test. Autofit of the PLS model revealed its good fitness (R^2^Y = 0.925) and predictive ability (Q^2^ value = 0.888). The permutation test presented in Fig. 4 indicated that the PLS model does not show overfitting, with the Y-intercepts of R^2^ and Q^2^ less than 0.3 and 0.05, respectively, and the R^2^ lines far from being horizontal [25]. These findings showed that the PLS model is valid and can appropriately correlate the identified phenolics with the studied bioactivities.

**Fig. 5:**
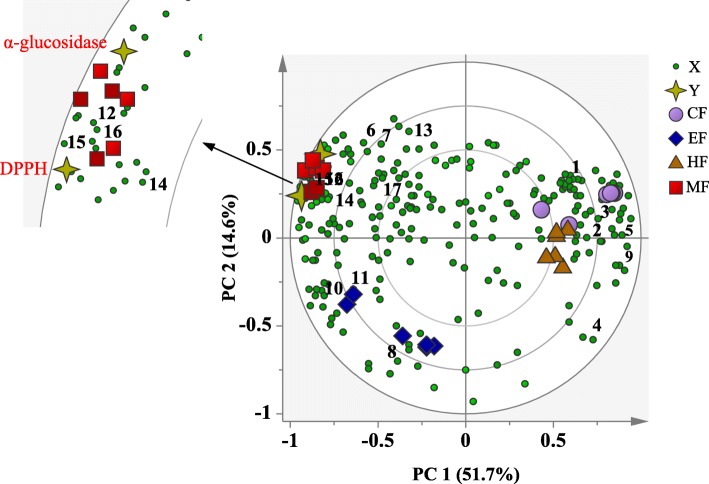
The PLS biplot of different fractions of *N. oleracea*. HF, hexane fraction; CF, chloroform fraction; EF, ethyl acetate fraction; MF, methanol fraction; 1, phytosterol; 2, fatty acids; 3, alanine; 4, leucine; 5, valine; 6, α-glucose; 7, β-glucose; 8, fructose; 9, oleanolic acid; 10, quercetin derivatives; 11, kaempferol derivatives; 12, apigenin derivatives; 13, myricetin derivatives; 14, gallic acid; 15, 3,4-*O*-dimethylgallic acid; 16, caffeic acid; 17, catechin

The PLS biplot is a combination of score and loading plots. Distance of the X and Y variables to the sample clusters indicates the extent of their contribution to the characteristics of the respective cluster. As shown in Fig. 5, the more polar fractions were separated from the less polar ones by the PC1, as previously observed in the PCA. Furthermore, the two Y variables (DPPH radical scavenging and α-glucosidase inhibitory activities) were projected on the same side with the EF and MF, indicating their better activities compared to those of the less polar fractions. Comparing the more polar fractions, the MF was more active than the EF, as the MF was closer to the Y variables. These results were in agreement with the bioactivities findings presented earlier (Fig. 3). In addition, all the identified phenolics were on the side where the active fractions located. This shows their positive contributions to the DPPH free radical scavenging and α-glucosidase inhibitory activities of the EF and MF. Among these phenolics, flavonoids including derivatives of quercetin and kaempferol play important roles in the free radical scavenging and α-glucosidase inhibitory activities of the EF; while derivatives of apigenin and phenolic acids were observed to be the important phytochemical markers for the MF.

**Fig. 6:**
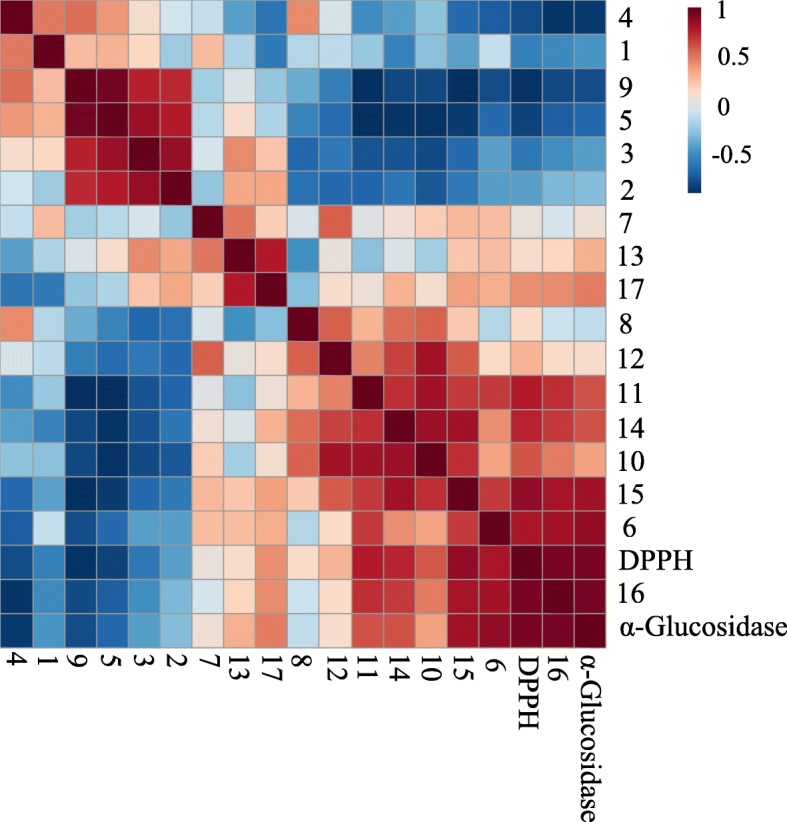
The overall Pearson’s correlation between the metabolites and bioactivities of different fractions of *N. oleracea*. The value of the correlation coefficients is represented by the blue and red colour intensities. 1, phytosterol; 2, fatty acids; 3, alanine; 4, leucine; 5, valine; 6, α-glucose; 7, β-glucose; 8, fructose; 9, oleanolic acid; 10, quercetin derivatives; 11, kaempferol derivatives; 12,apigenin derivatives; 13, myricetin derivatives; 14, gallic acid; 15, 3,4-*O*-dimethylgallic acid; 16, caffeic acid; 17, catechin

Pearson’s correlation was also performed to support the correlation of the metabolites with the DPPH and α-glucosidase inhibitory activities and the results are presented in Fig. 6. Pearson’s correlation showed positive correlations between the studied bioactivities and identified phenolics. This result demonstrates the relationship of these phenolics with the DPPH and α-glucosidase inhibitory activities of *N. oleracea.* Moreover, these metabolites had also high correlation with each other as well as with the sugars.

### UHPLC–MS/MS profiling of *Neptunia oleracea* bioactive fractions

**Fig. 7:**
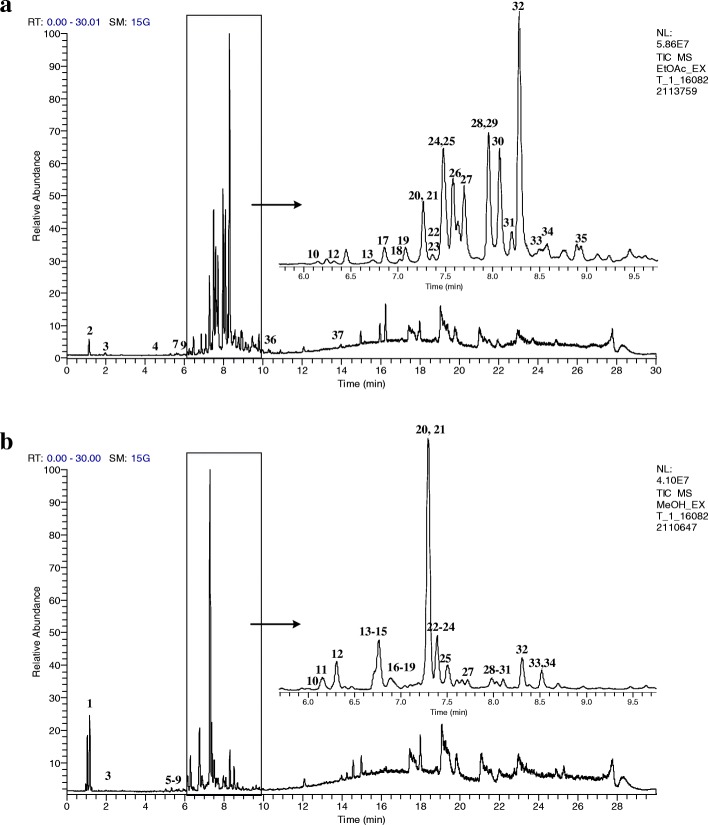
The total ion chromatogram of EF (**a**) and MF (**b**)

The EF and MF exhibited the most significant DPPH free radical scavenging and α-glucosidase inhibitory activities as the phenolic compounds were mainly distributed in these fractions. Hence, these fractions were subjected to UHPLC–MS/MS analysis to identify the derivatives of the phenolics present. The respective total ion chromatograms (TIC) of the EF and MF are presented in Fig. 7, while Table 2 summarizes the retention time (RT), MS/MS data and distribution of the identified metabolites. The TIC profiles showed that most of the prominent peaks were attributed to the presence of flavonoids. A total of 37 metabolites were tentatively identified based on the MS/MS data in comparison with the literature. These identified metabolites were classified into 7 groups, namely quercetin and its derivatives, apigenin derivatives, myricetin derivatives, kaempferol and its derivatives, other flavonoids, phenolic acid derivatives and other metabolites. Most of the identified metabolites were present in both the EF and MF. However, it was observed that the derivatives of phenolic acids as well as some flavonoid di- and triglycosides were only present in the MF, while the flavonoid aglycones were detected only in the EF.

**Table 2:** Identification of phenolic constituents in bioactive fractions of *N. oleracea* based on UHPLC–MS/MS data

Peak no.	Retention time (min)	[M-H]^−^	MS^2^ fragments	Metabolites	Fraction
Quercetin derivatives					
10	6.08	609.1462	462.1379, 447.1857, 301.1432, 300.9840	Quercetin 3-*O*-α-L-rhamnoside-7-*O*-β-D-glucoside	EF, MF
14	6.76	755.2040	489.2963, 343.1366, 301.0490, 300.0709, 271.2123, 254.9304, 179.0495, 169.3014	Quercetin 3-*O*-(2, 6-di-*O*-rhamnosylglucoside)	MF
15	6.81	625.1174	463.0464, 301.0478, 300.0883	Quercetin-3,7-di-*O*-glucoside	MF
22	7.36	609.1458	300.0327, 271.0090, 179.0178, 150.9577	Quercetin-3-*O*-rutinoside (Rutin)	EF, MF
26	7.70	477.0439	300.0253, 179.8799, 151.0737	Quercetin-3-*O*-glucoronide	EF
27	7.71	463.0919	300.070776, 271.1503, 255.2690, 179.0888, 151.0687	Quercetin-3-*O*-glucoside	EF, MF
28	7.94	433.0780	300.1089, 283.1775, 271.1692, 255.2084, 179.1739, 151.1124	Quercetin-3-*O*-xyloside	EF, MF
29	7.97	623.0173	315.0298, 314.0549, 300.0549, 151.0594	Isorhamnetin-3-*O*-rutinoside	EF, MF
30	8.07	433.0779	300.0705, 271.1375, 255.0952, 179.1469, 151.0447	Quercetin-3-*O*-arabinoside	EF, MF
32	8.29	447.0931	301.0434, 300.0367, 271.0428, 255.0371, 178.9992, 151.0714	Quercetin-3-*O*-rhamnoside	EF, MF
36	10.25	301.0394	273.0342, 178.9904, 151.0353, 121.0899	Quercetin	EF
Apigenin derivatives					
12	6.30	593.1517	473.0582, 383.2079, 353.1768, 297.0308	Apigenin-6,8-di-*C*- β-*D*-glucopyranoside (Vicenin 2)	EF, MF
21	7.31	577.1563	457.0943, 413.1237, 311.1826, 293.0732	Vitexin-2″-*O*-rhamnoside	EF, MF
23	7.39	431.1924	413.1062, 341.0215, 311.1169, 283.0694	Vitexin	EF, MF
24	7.45	431.0983	413.1362, 353.1137, 341.0769, 311.1139, 283.1790	Isovitexin	EF, MF
Myricetin derivatives					
16	6.88	625.1409	317.0156, 316.0339, 271.0775, 179.0016	Myricetin-3-*O*-rutinoside	MF
18	7.03	479.0832	316.0643, 287.0108, 271.0499, 179.0824, 151.0429	Myricetin-3-*O*-glucoside	EF, MF
19	7.07	463.0921	316.0347, 286.7651, 178.9603, 150.8803	Myricetin-3-*O*-rhamnoside	EF, MF
20	7.25	449.0729	316.0244, 287.0781, 271.0527, 179.0751	Myricetin-3-*O*-arabinoside	EF, MF
Kaempferol derivatives					
25	7.51	593.0462	473.0836, 447.1271, 327.0242, 285.0153, 284.0652	Kaempferol 7-*O*-(2″-rhamnosyl)-glucoside	EF, MF
31	8.22	447.1433	283.9436, 255.0079, 227.1132	Kaemperol-3-*O*-glucoside	EF, MF
34	8.52	417.1216	284.9989, 284.0469, 255.0592, 227.2742	Kaempferol-3-*O*-arabinoside	EF, MF
35	8.91	431.0986	285.0370, 284.0700, 255.0252, 227.0798, 179.1416	Kaempferol-3-*O*-rhamnoside	EF, MF
37	13.95	285.2077	285.1903, 217.0465, 151.1716	Kaempferol	EF
Other flavonoids					
8	5.77	609.0889	519.1772. 489.0393, 399.0961, 369.1139	Luteolin-6,8-di-*C*-β-*D*-glucopyranoside (Leucenin 2)	MF
9	5.97	289.0684	245.1160, 203.1322, 179.0933, 137.0067, 123.0869	Catechin	EF, MF
13	6.70	457.0777	331.6168, 304.8813, 287.1606, 269.0464, 192.9943, 169.0064	(−)-Epigallocatechin-3-gallate	EF, MF
17	6.85	447.0937	357.1292, 339.0839, 327.0893, 297.0871, 285.0973	Luteolin-8-*C*-glucoside (Orientin)	EF, MF
33	8.51	461.1090	446.0747, 297.9769, 283.0961, 255.3977	Gliricidin-*O*-hexoside	EF, MF
Phenolic acid derivatives					
2	1.20	304.9145	175.0625, 146.8956	Cinnamic acid derivative	EF
3	1.95	331.0674	312.9470, 271.0817, 210.9991, 168.9899, 151.9876, 125.0873	Monogalloylglucose	EF, MF
5	4.74	427.0858	358.9084, 197.0540, 182.0402, 153.2345, 138.0547	Syringic acid derivative	MF
6	5.31	285.0620	152.9958, 152.0484, 134.9576, 109.0381, 108.0353	Protocatechuic acid-*O*-pentoside	MF
7	5.51	483.0779	271.0838, 211.4571, 169.0153	Digalloylglucose	EF, MF
11	6.14	367.0671	183.0357, 168.1031, 139.0077, 123.9010	Methylgallate dimer	MF
Others					
1	1.17	683.2252	341.0159, 178.9980, 161.0347, 149.0118, 143.0902, 130.9740, 118.9781, 89.0819, 70.9360	Hexose polymer	MF
4	4.60	315.1091	153.0167, 123.0025	Hydroxytyrosol hexoside	EF

## Discussion

In the present study, ethanolic crude extract of *N. oleracea* was subjected to SPE to yield 4 fractions with different polarities (HF, CF, EF and MF). Metabolites from different classes were identified in the fractions. Both scrutiny of the ^1^H NMR spectra and unsupervised MVDA (PCA) showed that the HF and CF contained higher contents of phytosterol, oleanolic acid, amino acids and fatty acids; while EF and MF were characterized by their higher content of sugars and identified phenolics. The principle of “like-dissolves-like” can explain the distribution of the identified metabolites in the different fractions. The SPE of *N. oleracea* crude extract involved separating the metabolites relative to their solubility in the eluting solvent. The long hydrocarbon chain of fatty acids and the cyclic hydrocarbon structures of triterpene and phytosterol made these metabolites have low polarities [26]. Thus, these metabolites were more likely dissolved and eluted by the solvents with low polarities, such as hexane and chloroform. In contrast, the sugar attachment and the presence of a polar functional group, such as phenolic hydroxyl and carbonyl groups, make the phenolics polar and hence more soluble in solvents with relatively higher polarities, such as ethyl acetate and methanol. The better recovery of phenolics by these solvents compared to hexane and chloroform has also been reported previously [27, 28].

The EF and MF showed higher TPC and exhibited more potent DPPH free radical scavenging and α-glucosidase inhibitory activities compared to HF and CF (Fig. 3). The correlation findings revealed the contributions of phenolics towards the studied bioactivities. Via PLS analysis, derivatives of quercetin and kaempferol were observed to play important roles in the free radical scavenging and α-glucosidase inhibitory activities of the EF; while derivatives of apigenin and phenolic acids were the important bioactivity contributors of the MF. These results were in agreement with those in previous studies that revealed the significance of apigenin, quercetin and kaempferol derivatives as well as caffeic acid as the antioxidants and α-glucosidase inhibitors in *N. oleracea* [11, 12]. The potent antioxidant and α-glucosidase inhibitory activities of these metabolites can be explained by the presence and number of phenolic hydroxyl groups [29, 30]. Furthermore, ortho-dihydroxyl (catechol) substitution had been reported to impart high antioxidant and α-glucosidase inhibitory activities to molecules [31]. Hence, the presence of catechol moiety may contribute to the potent activities of these metabolites, particularly the quercetin derivatives and caffeic acid. In addition, Pearson’s correlation suggested synergistic effects of the phenolics towards the studied bioactivities due to their strong correlations with each other. Besides, the result revealed that the high intensity of phenolics was associated with the sugar present. This positive relationship between carbon-rich secondary metabolites such as phenolics and the sugar has also been reported previously [32, 33].

In order to identify the derivatives of phenolics present in the bioactive fractions, the EF and MF were subjected to UHPLC-MS/MS analysis. The UHPLC coupled with tandem MS represents a robust tool to characterize the metabolites in complex mixtures, owing to the combination of separation capability of UHPLC and identification ability of tandem MS. Together with some non-phenolic metabolites, a total of 37 metabolites were tentatively identified in the EF and MF. Most of these metabolites were present in both the EF and MF, with the exception of some metabolites which present in either one of the fraction, as shown in Fig. 7 and Table 2. This difference might be due to the different polarities of the metabolites, which were affected by the presence and number of the hydroxyl groups as well as sugar substitutions [34]. Identification of the metabolites by the UHPLC-MS/MS are discussed in detail.

### Identification of quercetin and its derivatives

In the EF and MF, a total of 10 metabolites were identified as quercetin derivatives based on the presence of an aglycone fragment ion at m/z 301 and the characteristic fragment ions at m/z 271 and 151 in their MS/MS spectra [35]. Metabolites **10** and **22** showed the same deprotonated molecular ion at m/z 609 but with a different pattern of fragmentation. Metabolite **10** was identified as quercetin 3-*O*-rhamnoside-7-*O*-glucoside based on the fragment ions at m/z 463, 447 and 301, which arose from the subsequent loss of rhamnose (m/z 146) and glucose (m/z 162) moieties. This fragmentation pattern was agreed with a previously reported pattern [36]. Meanwhile the fragment ions at m/z 463 and 447 were absent in metabolite **22**, showing less prominent fragmentation of this metabolite. This finding suggests that the rhamnose and glucose are probably attached together as a rutinose. This observation was similar to those reported by Sanchez-Rabaneda et al. [37]. Hence, metabolite **22** was assigned as quercetin-3-*O*-rutinoside (rutin).

The transition of the deprotonated molecular ion of metabolite **14** (m/z 755) to the quercetin ion (m/z 301) showed the loss of 2 rhamnosyl and 1 glucosyl moieties. This metabolite was identified as quercetin 3-*O*-(2,6-di-*O*-rhamnosylglucoside) by comparing it with reported MS/MS data [38]. Metabolite **15** gave a deprotonated molecular ion at m/z 625 and fragment ions at m/z 463 and 301, showing the loss of two glucose moieties. Hence, this metabolite was identified as quercetin-3,7-di-*O*-glucoside [39]. Metabolites **26**, **27**, **32** resulted in a quercetin fragment ion from the loss of glucuronyl (m/z 176), glucosyl (m/z 162) and rhamnosyl (m/z 146) moieties, respectively. Consequently, these metabolites were identified as quercetin-3-*O*-glucoronide, quercetin-3-*O*-glucoside and quercetin-3-*O*-rhamnoside, respectively [38, 40]. Metabolites **28** and **30** had both the same deprotonated molecular ion at m/z 433 and the same fragmentation pattern. However, based on the elution order and the previously reported data [41], metabolite **28** was assigned as quercetin-3-*O*-xyloside; while the later eluting metabolite **30** was assigned as quercetin-3-*O*-arabinoside. A methylated type of quercetin derivative was also detected. With the presence of a deprotonated molecular ion at m/z 623 and a fragment ion at m/z 315 due to the loss of rutinoside (m/z 308), metabolite **29** was identified as isorhamnetin-3-*O*-rutinoside [42]. Lastly, metabolite **36** was identified as quercetin based on the deprotonated molecular ion at 301 in its MS/MS spectrum [35].

### Identification of apigenin derivatives

Thalang et al. [43] had reported the presence of apigenin derivatives in *N. oleracea* extracts. However, the identity of these derivatives had not yet been revealed. In this present study, four derivatives of apigenin were tentatively identified in the EF and MF of *N. oleracea*. These metabolites showed the loss of m/z 120 and 90 in their MS/MS spectra, which are the typical fragmentation of *C*-glycoside that corresponding to the cross-ring cleavages in the sugar unit [37]. With the deprotonated molecular ion at m/z 593 and *C*-glycoside fragmentation pattern, metabolite **12** was identified as apigenin-6,8-di-*C*-glucoside (vicenin 2). This fragmentation behavior was consistent with the previous characterization [40]. Both metabolite **23** and **24** showed the same deprotonated molecular ion at m/z 431 and common characteristic fragment ions at m/z 341 (loss of m/z 90) and 311 (loss of m/z 120). However, after comparison of the elution order and with the previously reported data [37], metabolite **23** was assigned as apigenin-6-*C*-glucoside (vitexin) while metabolite **24** was assigned as apigenin-8-*C*-glucoside (isovitexin). The presence of the fragment ion at m/z 353 also differentiated isovitexin from vitexin, as reported by Sanchez-Rabaneda et al. [37]. Metabolite **21** showed a deprotonated molecular ion at m/z 577. The transition of m/z 577 to 413 showed the loss of a rhamnose unit, while the presence of the ion at m/z 457 (due to loss of m/z 120) revealed the cross-ring cleavage of the hexose moiety and the linkage between this hexose and the rhamnose is at the 1–2 position [44]. With this spectral evidence, metabolite **21** was elucidated as vitexin-2″-*O*-rhamnoside.

### Identification of myricetin derivatives

Metabolites **16**, **18**, **19** and **20** were identified as myricetin derivatives based on the presence of a fragment ion at m/z 316, corresponding to the myricetin aglycone fragment in the MS/MS spectra [35]. Metabolite **16** was identified as myricetin-3-*O*-rutinoside based on the transition of m/z 625 to 316, which revealed the loss of a rutinose moiety [45]. Metabolites **18**, **19** and **20** were assigned as myricetin-3-*O*-glucoside, myricetin-3-*O*-rhamnoside and myricetin-3-*O*-arabinoside based on the deprotonated molecular ions at m/z 479, 463 and 449, respectively. The transition of these ions to m/z 316 revealed the losses of the respective sugar moieties [38, 45].

### Identification of kaempferol and its derivatives

Four metabolites (**25**, **31**, **34** and **35**) were detected and assigned as kaempferol derivatives in the EF and MF. All these metabolites showed characteristic fragment ions at m/z 285 due to the loss of sugar moieties. The transition of m/z 593 to m/z 285 in metabolite **25** indicated the loss of the *O*-rhamanosyglucosyl moiety and the presence of the fragment ion at m/z 327 indicated the presence of a 1 → 2 isomer [46]. Therefore, metabolite **25** was identified as kaempferol 7-*O*-(2″-rhamnosyl)-glucoside. Meanwhile, the MS/MS data of metabolites **31**, **34** and **35** showed the losses of m/z 162, 132 and 146, which were characteristic of the presence of glucosyl, arabinosyl and rhamnosyl moieties, respectively. Hence, metabolites **31**, **34** and **35** were identified as kaemperol-3-*O*-glucoside, kaempferol-3-*O*-arabinoside and kaempferol-3-*O*-rhamnoside, respectively. These assignments were consistent with previously reported data [47]. Kaempferol aglycone (metabolite **37**) was detected in the EF with the presence of fragment ions at m/z 217 and 151 [37].

### Identification of other flavonoids

Besides the derivatives of quercetin, kaempferol, myricetin and apigenin, derivatives of other flavonoids were also detected in the EF and MF of *N. oleracea*. Metabolites **8** and **17** were detected and elucidated as *C*-glycosides of luteolin based on the losses of m/z 90 and 120. Comparison of the spectral data with those reported had identified metabolite **8** and **17** as luteolin-6,8-di-*C*-glucoside (leucenin 2) and luteolin-8-*C*-glucoside (orientin), respectively [48]. Catechin and its derivative were also detected. Metabolite **9** showed a deprotonated molecular ion at m/z 289 and yielded fragment ions at m/z 245, 179 and 137, which matched with those reported for catechin [49]. Hence, metabolite **9** was assigned as catechin. Meanwhile, metabolite **13** yielded fragment ions m/z 331 and 169, which was corresponding to the epigallocatechin and gallic acid moieties, respectively. Subsequently, metabolite **13** was identified as (−)-epigallocatechin-3-gallate [50]. Metabolite **33** showed a deprotonated molecular ion at m/z 461. The MS/MS data showed the loss of a hexose moiety and the subsequent fragmentation pattern of gliricidin, based on comparison with previously described characterization [51]. Hence, metabolite **33** was identified as gliricidin-*O*-hexoside.

### Identification of phenolic acid derivatives

Derivatives of phenolic acids were also detected and identified as well. Analysis of the deprotonated molecular ions at m/z 331 and 483 (metabolites **3** and **7**, respectively) showed the presence of galloylglucose. In the fragmentation of ion m/z 331, the loss of a glucose moiety yielded the fragment ion m/z 169, which originated from a gallic acid. Hence, metabolite **3** was identified as monogalloylglucose [52]. Metabolite **7** was assigned as digalloylglucose based on comparison of the fragmentation pattern with those reported [52]. Metabolite **2** and **5** yielded fragment ions at m/z 147 (cinnamic acid moiety) and m/z 197 (syringic acid moiety), and they were identified as unknown derivatives of cinnamic and syringic acids, respectively. Metabolite **6** was identified as protocatechuic acid-*O*-pentoside, by the ions m/z 153, 135 and 109, which originate from protocatechuic acid and its subsequent fragmentation [53]. Metabolite **11**, with a deprotonated molecular ion at m/z 367 and fragmentation data that corresponding to methylgallate, was identified as a dimer of methylgallate [53].

### Identification of other metabolites

A derivative of phenylethanoid was also detected in the EF. Metabolite **4** was identified as hydroxytyrosol hexoside as the transition of the deprotonated molecular ion at m/z 315 to the fragment ion of hydroxytyrosol at m/z 153 showed the loss of a hexose moiety [54]. In addition to all the aforementioned phenolic constituents, an oligosaccharide was identified. Metabolite **1**, detected in the MF was identified as a hexose polymer, as the fragment ions at m/z 341 and 179 revealed the losses of hexose moieties. Besides, the fragment ions at m/z 143, 131, 119, 113, 89, and 71 are the typical fragments that arise from hexose [55], thus further confirming the presence of hexose.

## Conclusions

*Neptunia oleracea* had been fractionated into 4 fractions with different polarities (HF, CF, EF and MF). The correlation study identified the EF and MF as the bioactive fractions of *N. oleracea* with regards to the DPPH scavenging and α-glucosidase inhibitory activities. Both activities showed strong correlation with the identified phenolics. Via the UHPLC-MS/MS profiling of the EF and MF, a total of 37 metabolites, with mostly phenolics were tentatively identified. Most of these identified metabolites were present in both the EF and MF. To the best of our knowledge, the differentiation, metabolite-bioactivity correlation and UHPLC-MS/MS profiling of bioactive fractions were performed for the first time on *N. oleracea*. The findings in this work are comprehensively supporting our previous work regarding the contribution of phenolics to the DPPH free radical scavenging and α-glucosidase inhibitory activities of this plant, with more detailed identification of these bioactive metabolites. In summary, *N. oleracea* is a rich source of phenolics that can be potential antioxidants and α-glucosidase inhibitors used in the management of diabetes.

## Abbreviations

CF: Chloroform fraction
EF: Ethyl acetate fraction
HF: Hexane fraction
MF: Methanol fraction
ROS: Reactive oxygen species
RT: Retention time
SPE: Solid phase extraction
TIC: Total ion chromatograms
TPC: Total phenolic content
UHPLC-MS/MS: Ultra-high performance liquid chromatography tandem mass spectrometry analysis
